# Effect of Protein Intake on Growth and Nutrient Retention of Pacific White Shrimp (*Litopenaeus vannamei*) in a Green Water Recirculating Aquaculture System

**DOI:** 10.1155/anu/4942439

**Published:** 2025-03-25

**Authors:** Adela N. Araujo, Khanh Nguyen, Leila Strebel, Trenton Corby, Melanie A. Rhodes, Benjamin H. Beck, Luke A. Roy, D. Allen Davis

**Affiliations:** ^1^School of Fisheries, Aquaculture, and Aquatic Sciences, Auburn University, Auburn, Alabama 36849, USA; ^2^Aquatic Animal Health Research Unit, USDA-ARS, Auburn, Alabama 36832, USA

**Keywords:** feed management, growth performance, nutrient intake, shrimp

## Abstract

Feed management and the nutrient content of the diet are two of the most important factors in shrimp culture, as feed makes up around 60% of the total variable costs. Given the cost of protein and its effect on growth, it is critical to understand the effects of both dietary protein and feeding rates on shrimp performance. Hence, the aim of this study was to evaluate the effect of different protein intake levels on growth performance, nutrient retention efficiency, and whole-body composition of Pacific white shrimp (*Litopenaeus vannamei*) using different levels of crude protein (CP). This was done by feeding four practical diets with 40%, 35%, 30%, and 25% of CP, which were fed at two different rates, one of them being the standard feed rate (100%) and a second adjusted rate to match the protein supplied (40% protein equivalence), resulting in a total of eight treatments with four replicates each. The total ration for each of the treatments was spread out into four feedings per day. Juvenile shrimp (0.41 ± 0.01 g) were stocked into a green water outdoor recirculating system with 32 circular tanks at a density of 30 individuals/tank and reared for 11 weeks. A recirculating aquaculture system (RAS) with green water (~30°C) was used for this trial, which consisted of a central reservoir (⁓1000 L) and treatment tanks (⁓800 L). At the end of the trial, growth performance parameters including final weight, weight gain (WG), biomass, and feed conversion ratio (FCR) were found to be significantly different among treatments (*p*  < 0.05). Average survival was between 94% and 98% for all the treatments. All final body composition values (dry matter, CP, and minerals) did not show significant differences between treatments except for fat (*p*  < 0.05). However, feed utilization measurements including apparent net protein retention (ANPR), and phosphorus retention (PR) showed to have significant differences (*p* < 0.01), ranging 49%–66% and 16%–27%, respectively. The results from this study demonstrated that treatments with higher protein intake resulted in the best growth performance, meanwhile treatments with lower protein intake had the highest nutrient retention values.

**Summary**

• Nutrient retention is directly correlated with the amount of nutrient supplied by both diet content and feeding ration.
• By increasing the feeding ration, diets of different CP levels can achieve the same growth performance but decreases in cost efficiency as the CP level in the diet decreases.
• Different rations of the same CP level diet had notable differences in size and growth, hence, the importance of feeding adequate rations.

## 1. Introduction

Aquaculture is a growing industry that is trying to help meet the protein demand of a continuously increasing population [1]. Since it is an industry in constant development, much still needs to be done to make it more efficient, sustainable, and profitable. A considerable proportion of aquaculture production is comprised by the shrimp farming industry, which has grown continuously since the 1980s due to major technological development, high demand, and public support [2, 3]. Within the United States, shrimp makes up approximately $10 million of the total value of marine species produced [4]. On a global scale, shrimp produced through aquaculture contributed 47.3% of the world's aquatic animal production for human consumption [5]. Hence, the culture of shrimp is a major contributor to the world seafood supply and consequently must continually look to increase efficiency and reduce costs [6].

In the case of Pacific white shrimp (*Litopenaeus vannamei*), feed costs represent approximately 68% of the total variable costs of culture expenses [7]. Protein is considered the costliest ingredient and an important nutrient restrain for growth in shrimp [8]. Furthermore, crude protein (CP) content in feed not only affects feed costs, but it can also increase the amount of nitrogen excreted by the shrimp that enters the culture system [9, 10]. While many studies have investigated the protein requirements for the optimal growth of this species and its effect on water quality, there remains considerable interest in culturing shrimp feeds at lower protein levels that still accounts for the basic requirements and results in suitable production parameters.

Presently, there is a belief among some commercial producers that the use of higher protein levels will translate into faster growth and better digestibility [11]. However, this may not always be true, especially in production systems with natural productivity. Having high protein feed in high productivity water may be counterproductive, as nitrogen loading negatively impacts water quality and wastewater released back into ecosystems [12].

According to studies [13, 14], proper feed management can often reduce feed costs by 15%–20%. Shrimp are continuous and slow feeders, which is why both feeding frequency and level are important topics in research and commercial production. It has been found that four to six feedings per day will make significant improvements in growth performance compared to only one to two feedings per day [15, 16]. This is because multiple portioned feedings results in a reduction of nutrient leaching loss [17]. Besides feeding frequency, feeding rations or rates also play an essential role.

There is an often overlooked interaction between nutrient density and feeding rations. It has been demonstrated that different rations of the same diet can have a significant impact on the growth performance of a variety of species [18–21].

Given there are several implications that can affect the way shrimp are able to leverage from nutrient in feed, the objective of this study was to evaluate the effect of shrimp growth and feed utilization when different levels of CP are offered at different feeding rates in an outdoor green water system.

## 2. Materials and Methods

### 2.1. Postlarvae (PLs) Nursing

Postlarval *L. vannamei* were sourced from Homegrown Shrimp LLC (Indiantown, FL) and transported to Claude Peteet Mariculture Center (CPMC) operated by the Alabama Conservation Department of Conservation and Natural Resources in Gulf Shores, AL, USA. PLs were then acclimated in six 6000 L outdoor tanks at a density of ~ approximately 22 PLs/L. These PLs were offered commercial diet including PL Raceway Plus 1, 2, 3 and PL Raceway 40-9 (Zeigler Bros, Inc., Gardners, PA, USA). PLs were attained until they reached an appropriate size and then transferred to the research system.

### 2.2. Experimental Design

At the beginning of the experiment, juvenile shrimp, averaging a weight of 0.41 ± 0.01 g (mean ± SD) were stocked in 32 tanks within an outdoor recirculating system, at a density of 35 shrimp/m^2^ (30 individuals per tank). Upon stocking, an initial sample of shrimp was also taken for whole-body composition analysis. The trial utilized a recirculating aquaculture system (RAS) with green water, comprising a central reservoir (~1000 L), circular treatment tanks (around 800 L each), and a 1/3 horsepower circulation pump facilitating a daily 5.0% water exchange rate. The green water was naturally procured from an outdoor pond system, drawing water from the intercoastal canal. The water was pumped from one outdoor shrimp production pond using a sump pump. Tanks were randomly assigned to one out of eight treatments.

### 2.3. Experimental Diets

Diets were formulated to include soybean meal and poultry by-product meal as the primary protein source (Table 1) to create four practical diets with different CP contents (25%, 30%, 35%, and 40%). The various protein levels were achieved by systematically reducing the primary protein source. Diets were manufactured under typical commercial conditions by Zeigler Bros. Inc. (ZBI, Gardners, PA, USA). Proximate composition (Table 1) and amino acid profile (Table 2) was carried out by the University of Missouri Agricultural Experiment Station Chemical Laboratories, according to established techniques and standard operating procedures.

### 2.4. Feed Management Treatment

Shrimp were fed four practical diets at two different rates, making up a total of eight different treatments. To match the protein supplied by the 40% CP diet at 100% standard feeding protocol (SFP), diets with lower CP (35%, 30%, and 25%) had an established an adjusted ration (114.3%, 133.3%, and 160%; Table 3). All diets were also fed at 100% SFP, and the 40% CP diet was reduced (87.5%) to match a protein equivalence of 35%. Experimental diets were offered four times a day to four replicate tanks. The standard feeding ration was calculated by using the predicted growth of the shrimp (doubling in weight after the first week) and a feed conversion ratio (FCR) of 1.2, based on the historic results of previous trials. Then, the standard ration was adjusted by multiplying it by the percentages previously stated. A subsample of shrimp was measured for each treatment biweekly to adjust the predicted growth using the standard ration calculation.

### 2.5. Water Quality

Dissolved oxygen (DO), temperature, and salinity parameters were monitored twice per day using a hand-held YSI. Total ammonia nitrogen (TAN) and pH were measured once per week using an ion-selective electrode for TAN, and the Orion 4-Star Plus pH/ISE (Thermo Fisher Scientific, Waltham, MA, USA) and the EcoScence pH10A (YSI, Yellow Springs, OH, USA) for pH. Weekly, a spectrophotometer (WaterLink spintouch LaMotte, Chestertown, MD, USA) was used to measure pH, ammonia, nitrite, nitrate, magnesium, calcium, phosphate, and alkalinity. All water quality parameters were maintained within a suitable range for culture of shrimp (Table 4).

### 2.6. Growth Performance

At the end of the 11-week growth trial, the final biomass was determined, and shrimps were enumerated. Mean weight (MW), weight gain (WG) (as % and in g), and FCR were calculated as follows:

  $$\begin{array}{c} \text{MW}=\frac{\text{Biomass }\left(\text{g}\right)}{\text{Number of shrimp}}. \end{array}$$

  $$\begin{array}{c} \text{WG }\left(\%\right)=\left(\left(\text{Final weight }\left(\text{g}\right)-\text{initial weight }\left(\text{g}\right)\right)|/|\text{initial weight }|\left(\text{g}\right)\right)\times 100\%. \end{array}$$

  $$\begin{array}{c} \text{WG}=\left(\text{Final weight }\left(\text{g}\right)-\text{initial weight }\left(\text{g}\right)\right). \end{array}$$

  $$\begin{array}{c} FCR=\frac{\text{Total feed }\left(\text{g}\right)}{\text{Final biomass}-\text{initial biomass}}. \end{array}$$

### 2.7. Nutrient Retention

At the end of the growth trial, a sample of five shrimp was taken from each tank for whole-body proximate composition analysis. The shrimp were dried, homogenized, and sent to Midwest Laboratories Inc. (Omaha, NE, USA). Apparent net protein retention (ANPR) and phosphorus retention (PR) were calculated as follows:

  $$\begin{array}{c} \text{ANPR}=\frac{\left(\left(\text{Final CP content }\left(\%\right)\times \text{final MW }\left(\text{g}\right)\right)|-|\left(\text{initial CP content }\left(\%\right)\times \text{initial MW }\left(\text{g}\right)\right)\right)}{\text{Protein offered }\left(\text{g}\right)}. \end{array}$$

  $$\begin{array}{c} PR=\frac{\left(\left(\text{Final Phosphorus content }\left(\%\right)\times \text{final MW }\left(\text{g}\right)\right)|-|\left(\text{initial Phosphorus content }\left(\%\right)\times \text{initial MW }\left(\text{g}\right)\right)\right)}{\text{Phosphorus offered }\left(\text{g}\right)}. \end{array}$$

### 2.8. Statistical Analysis

All data were analyzed using SAS Version 9.4 (SAS Institute, North Carolina, USA) and an alpha value of *α* = 0.05. To compare results among treatments, an analysis of variance (ANOVA) was used and significant outcomes were tested between treatment means using a post hoc test, the Tukey's honest significant difference for multiple comparisons.

## 3. Results

### 3.1. Water Quality

Water quality was maintained within acceptable ranges throughout the course of the experiment (Table 4). Survival rates between treatments were not significantly different (*p*  > 0.05) and all treatments showed good survival ranging from 94% to 98% (Table 5).

### 3.2. Growth Performance

**Figure 1:**
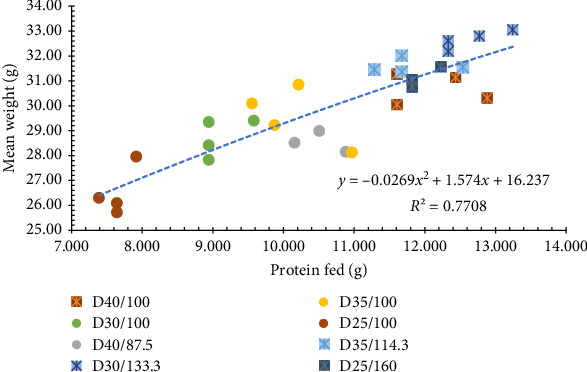
Final mean weight (MW) of Pacific white shrimp reared in a green water recirculating system where juveniles were stocked at 35 shrimp/m^2^ and fed four protein variable diets (D25-40%) over the calculated protein fed according to the amount of feed given for each of the treatments.

**Figure 2:**
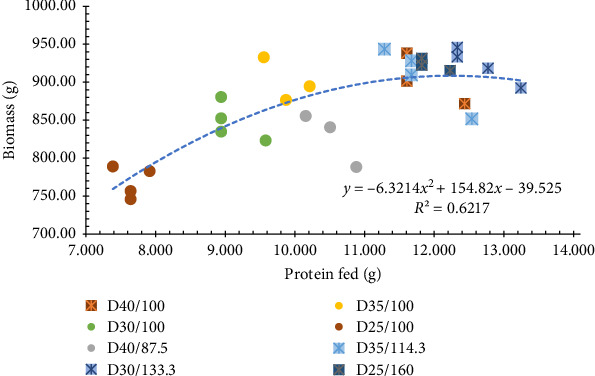
Final biomass of Pacific white shrimp reared in a green water recirculating system where juveniles were stocked at 35 shrimp/m^2^ and fed four protein variable diets (D25-40%) over the calculated protein fed according to the amount of feed given for each of the treatments.

All growth performance parameters resulted in a significant difference (*p*  < 0.05) among treatments. Shrimp fed a higher ration had improved in WG, MW, and yield. The treatments fed at 100% ration decreased in growth performance as the content of CP in the diet decreased, meanwhile diets at higher rations (all 40% protein equivalence) had similar results (Table 5). When observing the results of each parameter individually in a graphical manner, they all indicated that treatments with 40% protein equivalence had the best growth performance (Figures 1 and 2). This matches the expected outcome, explaining that it is possible to have the same results in terms of growth on a lower CP diet by managing the daily intake. Diets with 30% and 35% CP, fed at the higher ration, had the best result for individual weight and biomass.

**Figure 3:**
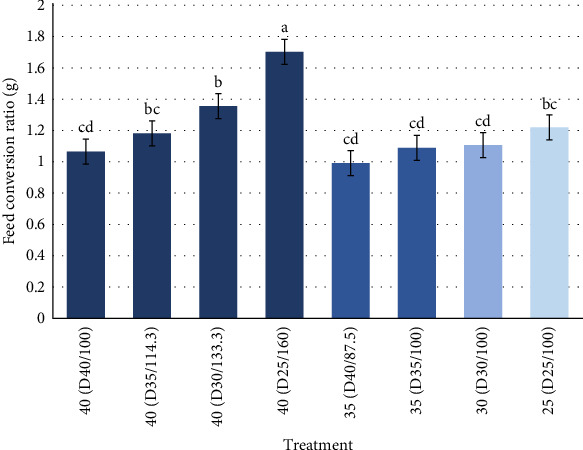
Feed conversion ratio (FCR) of Pacific white shrimp reared in a green water recirculating system where juveniles were stocked at 35 shrimp/m^2^ and fed four protein variable diets (D25-40%) over a 11-week period. Shades of blue determine different protein equivalence for each of the treatments. Different letters (a, b, c, d) represent statistical differences based on Tukey's test (*p* < 0.05, one-way ANOVA).

In the case of FCR, as expected, we can see that higher ration with the lowest CP levels had the highest FCR and reduced rations with the high protein content had the lowest FCR amongst the treatments (Table 5). It is important to point out that when using higher rations with lower CP levels, the FCR significantly increased (Figure 3). Indicating that more feed is needed so that the animals can grow to the same size and to compensate for the protein requirements. Whereas for the treatments who were fed according to standard ration (100%) and lower rations, it can be observed that these had lower FCR values and did not have any statistical differences.

**Figure 4:**
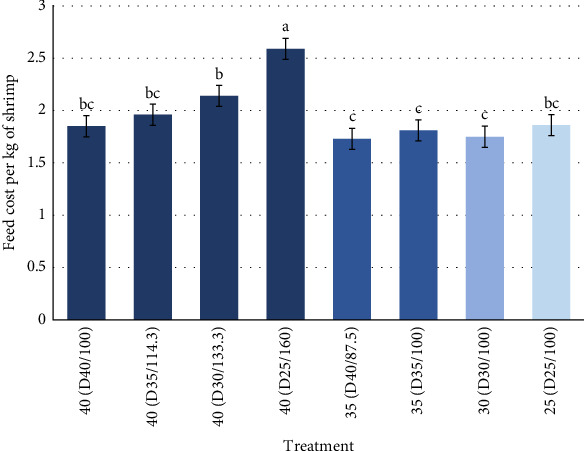
Feed cost per kg of Pacific white shrimp reared in a green water recirculating system where juveniles were stocked at 35 shrimp/m^2^ and fed four protein variable diets (D25-40%) over a 11-week period. Shades of blue determine different protein equivalence for each of the treatments. Different letters (a, b, c) represent statistical differences based on Tukey's test (*p* < 0.05, one-way ANOVA).

Treatments were significantly different from each other in terms of feed cost (Table 5). Higher rations and lower protein contents were the most expensive to produce 1 kg of shrimp. As seen in Figure 4, for feed cost, the treatments with 40 protein equivalence increased in cost as the ration increased. Based on this results, as the ration increases consequentially the profit will decrease. For treatments at 100% standard ration, we see no difference in cost, but this does not account for the fact that these are at different protein intake resulting in shrimp with different sizes (and therefore value).

### 3.3. Whole-Body Composition and Nutrient Retention

For proximate composition analysis, all values shown as treatment means are presented in Table 6. Aside from fat, all whole-body composition variables including moisture, protein, fiber, ash, sulfur, potassium, magnesium, calcium, sodium, iron, manganese, copper, and zinc showed no significant difference between treatments. Particularly for fat, the 25% CP diet at a standard feeding ration had the lowest value for fat compared to all other treatments (*p*  = 0.014).

**Figure 5:**
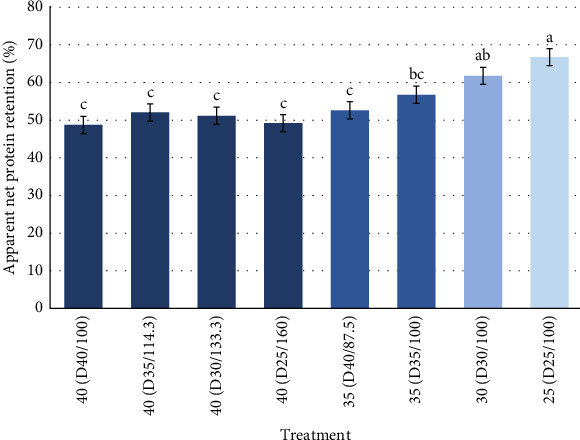
Apparent net protein retention (ANPR) of Pacific white shrimp reared in a green water recirculating system where juveniles were stocked at 35 shrimp/m^2^ and fed four protein variable diets (D25-40%) over a 11-week period. Shades of blue determine different protein equivalence for each of the treatments. Different letters (a, b, c) represent statistical differences based on Tukey's test (*p* < 0.05, one-way ANOVA).

ANPR means (*p*  < 0.05) for each treatment demonstrated that low rations with reduced CP content developed higher protein retention (Table 5). As protein intake decreased, the apparent net protein increased. Conversely, higher protein intakes with different increased rations maintained approximately the same range in ANPR value. ANPR results are presented graphically in Figure 5. As is typically reported, protein retention was improved in treatments that had lower protein intake. It also shows that for the higher protein intake, treatments had the lowest protein retention, and they were all closely in the same range.

**Figure 6:**
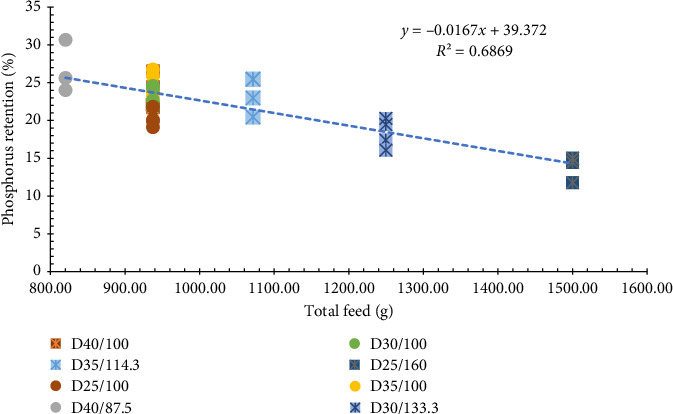
Phosphorus retention (PR) of Pacific white shrimp reared in a green water recirculating system where juveniles were stocked at 35 shrimp/m^2^ and fed four protein variable diets (D25-40%) for 11 weeks.

Last, PR was also significantly different between treatments. This nutrient also showed a clear trend, as lower rations resulted in higher retention rates. PR also showed that there is a negative relationship between the feeding ration and PR (Figure 6).

## 4. Discussion

### 4.1. Growth Performance

There is an obvious need for an increase in shrimp culture worldwide to be able to meet the market demand. At the same time, the shrimp industry is being set back by high feed prices and inefficient feeding practices. As stated by previous studies, protein requirements for white leg shrimp could range from 25% to 40% [8, 22, 23]. These recommendations are based on a range of factors which influence the perceived requirements. These factors include nutrient balance, digestibility, genetic potential for growth, and environment, as well as feed management. To meet nutrient requirements, one must have an adequate daily intake of that nutrient; hence, there is a clear interaction between nutrient density and feed management or daily intake [24–26]. In terms of protein requirements, it has been demonstrated that lower protein content with high consumption may result in similar growth as high protein diets with lower consumption [19, 25, 27]. The results of this study expand on previous studies looking at feed intake and protein content concerning growth performance and nutrient retention.

The FCR was between the acceptable ranges for shrimp reared in systems with natural productivity [28] and would indicate the even at higher feed inputs the feed was consumed (Figure 1). As the FCR is directly dependent on the amount of nutrients consumed [29], our results corroborate the fact that higher rations of lower nutrient dense diets will result in less efficient production. The results from this study demonstrate that shrimp offered the standard ration had less efficient growth performance as the protein content in the diet decreased (Figures 1 and 2). Demonstrating the importance of having protein content and feed intake in mind when choosing what and how much to feed.

Feed cost was also a key factor to consider for this study, as costs play an important role when considering the amount of feed being used daily. As would be expected, the protein content of the diet increased and so did the cost per unit weight; however, the cost per unit protein decreased (Table 1). The results of this trial showed that at a fixed feed input (100% ration) that there was not much difference in feed cost/kg shrimp; however, under these conditions, the growth of shrimp on lower protein diets is considerably slower meaning more days for a given market size which increased the overall production costs in multi-batch systems. When comparing these costs with similar growth rates (i.e., shrimp fed similar protein equivalence), it resulted in very different feed costs per kg of shrimp, which increased with decreasing dietary protein level. Despite the fact that we can get the same growth with lower protein diets by adjusting the ration, it may not always be beneficial economically.

### 4.2. Whole-Body Composition and Nutrient Retention

Shifting the protein content of the diet could also influence the proximate composition of the shrimp; hence, whole-body compositions were analyzed. We did not see any difference in the nutrients found in the shrimps' whole body, except for fat. Seeing that the lowest was 25% at a 100% standard ration, this could be explained with the fact that the shrimps with this treatment were being underfed protein and energy as growth was reduced. Consequently, they were deficient in lipids or energy stores. After identifying no major differences in whole-body composition values, the nutrient retention indicators were further examined.

In terms of protein retention, the results of this study (Figure 5) are similar to those of Strebel et al. [30] and Weldon et al. [27] that demonstrated the same trend in Pacific white shrimp with varying feeding rates in a biofloc system and an outdoor pond system, in which lower rates or daily intake of feed had the highest protein retention. This outcome was attributed to the presence of natural foods, which also matches the conditions of this growth trial. In general, ANPR values are reduced as feed inputs and/or nutrient intake increase, as growth is increased and consequently the contribution of natural productivity is reduced. Yaemsooksawat et al. [31] also states that as the protein level increases, protein efficiency decreases.

Another possible explanation is that when given more protein, the shrimp retains it in the tissue up to a certain point and after that it will start using it as an energy source resulting in less efficient deposition. It has also been widely proven in multiple species (such as swine and poultry) that when given less of a nutrient (in this case protein) than the required level, the organism will try to retain more and draw upon it for growth [32, 33]. Interestingly, at a fixed protein intake (Figure 5), there were no differences of protein retention indicating at the same protein intake and similar growth rates protein retention is similar.

As opposed to protein, the content of phosphorus (P) in the diet was not controlled, so the levels ranged from 6.0 to 8.0 (g/100 g of feed). Hence, any increase in feed input resulted in an increase in P intake and a reduction in protein retention. Since the diets were not adjusted to have different levels of P, this result makes sense. When offered less than required, shrimp should have a higher retention of the nutrient.

## 5. Conclusion

Under the experimental conditions examined in this trial, protein intake, whether it was through diet formulation or through a feeding ration, significantly affects the growth and nutrient retention performance of *L. vannamei*. Higher protein intake resulted in better growth performance of shrimp, but diet cost and daily ration should also be considered when comparing feed management strategies. When feeding in higher quantities/rations, the more expensive it is, so it may not be cost effective to offer shrimp a low protein diet. On the other hand, use of a high protein diet throughout the production cycle for large production systems may also present long-term economic challenges, as well as pose a higher amount of environmental risk in terms of increased waste production. Based on the results of this trial, shrimp diets containing 30%–35% CP appear to be the most suitable alternative for commercial producers seeking to balance both performance and cost.

## Figures and Tables

**Table 1:** Formulation (g/100 g as is) of four diets formulated to contain 25%, 30%, 35%, and 40% protein and 5%, 6%, 7%, and 8% lipid which were commercially extruded (Zeigler Bros, Inc. Gardners, Pennsylvania, USA) as a 2.4 mm sinking feed.

Ingredient/nutrient	D25	D30	D35	D40
Soybean meal (47.5% protein)	33.00	43.00	49.00	57.00
Whole wheat	52.10	37.60	27.93	15.23
Poultry-by meal (67% protein)	2.00	4.00	6.00	8.00
Corn gluten meal (60% protein)	2.00	4.00	6.00	8.00
Dicalcium phosphate	3.13	3.13	2.00	2.00
Fish oil	3.00	3.50	4.30	4.00
Squid meal	2.00	2.00	2.00	2.00
Bentonite	1.50	1.50	1.50	1.50
Lecithin	1.00	1.00	1.00	1.00
Vitamin premix^a^	0.12	0.12	0.12	0.12
Mineral premix^a^	0.12	0.12	0.12	0.12
Stable C (35% active)	0.02	0.02	0.02	0.02
Copper sulfate	0.01	0.01	0.01	0.01

CP	26.55	31.99	32.77	40.57
Moisture	8.44	7.52	8.35	7.75
Crude fat	6.10	7.54	8.53	8.72
Crude fiber	2.61	3.12	3.00	2.84
Ash	7.45	7.93	7.3	7.65
Cu	101	95.4	102	114
P	1.26	1.25	1.11	1.20

Cost ($/kg)	1.52	1.58	1.66	1.74

Cost per unit of protein ($/g)	0.015	0.016	0.017	0.017

Abbreviations: CP, crude protein; P, phosphorus.

^a^Vitamin and mineral premixes are proprietary products, and therefore, the composition is not listed.

**Table 2:** Amino acid profile analysis of four protein variable diets (25%–40%) offered at two different rations to *Litopenaeus vannamei* cultured in a greenwater system for 11 weeks.

Amino acid	D25	D30	D35	D40
Alanine	1.23	1.53	1.75	2.10
Arginine	1.67	2.06	2.28	2.70
Aspartic acid	2.43	3.05	3.42	4.04
Cysteine	0.41	0.46	0.49	0.56
Glutamic acid	5.11	5.91	6.48	7.40
Glycine	1.24	1.48	1.65	1.96
Histidine	0.64	0.77	0.86	1.01
Hydroxy lysine	0.02	0.02	0.03	0.03
Hydroxy proline	0.23	0.25	0.27	0.28
Isoleucine	1.13	1.37	1.54	1.81
Lanthionine §	0.04	0.04	0.05	0.07
Leucine	2.07	2.57	2.95	3.47
Lysine	1.48	1.79	2.00	2.36
Methionine	0.43	0.50	0.57	0.66
Ornithine §	0.03	0.03	0.03	0.05
Phenylalanine	1.28	1.55	1.75	2.04
Proline	1.56	1.80	1.98	2.29
Serine	1.14	1.38	1.51	1.75
Taurine §	0.22	0.19	0.17	0.17
Threonine	0.96	1.17	1.32	1.55
Tryptophan	0.33	0.38	0.41	0.46
Tyrosine	0.94	1.17	1.31	1.54
Valine	1.23	1.47	1.65	1.96

*Note:* Results are expressed as g/100 g as is.

**Table 3:** Dietary treatments of four experimental diets being offered at two different rations.

Crude protein (%)	% Standard ration (%)	Protein equivalence
40	100	40
35	114.3	40
30	133.3	40
25	160	40
40	87.5	35
35	100	35
30	100	30
25	100	25

*Note:* For the first four treatments rations were adjusted to have the same protein equivalence.

**Table 4:** Summary of water quality parameters in a green water RAS system throughout 11 weeks of the trial duration.

DO (mg/L)	6.92 ± 1.19 (3.73, 11.73)
Temperature (°C)	30.40 ± 2.52 (25.3, 35.5)
Salinity (g/L)	9.81 ± 2.40 (3.09, 14.38)
TAN*⁣*^*∗*^ (mg/L)	0.27 ± 0.30 (0, 1.0)
Nitrite nitrogen (mg/L)	0.53 ± 0.74
Nitrate nitrogen (mg/L)	0.45 ± 0.52
pH	7.26 ± 0.38
Phosphate (mg/L)	2.67 ± 1.97
Calcium (mg/L)	135.09 ± 42.18
Magnesium (mg/L)	341.27 ± 94.08
Alkalinity (mg/L)	43.36 ± 9.50

*Note:* Values are shown as the mean ± standard deviation and the minimum and maximum values below in parenthesis for DO, temperature, salinity, and TAN.

Abbreviations: DO, dissolved oxygen; RAS, recirculating aquaculture system; TAN, total ammonia nitrogen.

*⁣* ^*∗*^Average ammonia done with an ion selective probe and a WaterLink spin touch FF tester.

**Table 5:** Growth performance and nutrient retention of Juvenile shrimp (~0.41 ± 0.01 g) stocked at density of 30 shrimp per tank (35/m^2^) and reared with different levels of protein intake under semi-intensive conditions in tanks of a green water RAS system for 77 days.

Protein/ration	Weight (g)	Survival (%)	Gain (g)	Biomass (g)	FCR	ANPR (%)	PR (%)	Feed cost per kg of shrimp ($)
D40/100%	30.69^abc^	95.83	30.27^ab^	882.45^ab^	1.08^cd^	48.72^c^	26.18^ab^	1.85^bc^
D40/87.5%^1^	28.55^cd^	96.66	28.14^bc^	828.07^ab^	1.01^d^	52.62^c^	29.57^a^	1.73^c^
D35/100%	29.57^bc^	97.5	29.16^ab^	865.85^ab^	1.11^cd^	56.71^bc^	27.32^ab^	1.81^c^
D35/114.3%	31.59^ab^	95.83	31.17^ab^	908.30^a^	1.20^bc^	52.03^c^	25.80^ab^	1.96^bc^
D30/100%	28.75^dc^	98.33	28.35^bc^	847.70^ab^	1.12^cd^	61.74^ab^	25.19^ab^	1.75^c^
D30/133.3%	32.66^a^	94.17	32.25^a^	922.50^a^	1.37^b^	51.17^c^	19.77^cd^	2.14^b^
D25/100%	26.52^d^	96.67	26.11^c^	768.55^b^	1.24^bc^	66.73^a^	23.63^bc^	1.86^bc^
D25/160%	30.26^bc^	97.5	29.86^ab^	886.15^a^	1.73^a^	49.21^c^	16.43^d^	2.59^a^
PSE	0.9345	0.404	0.9352	48.02	0.0792	3.65	2.169	0.0872
*p*-value^2^	<0.001	0.8778	<0.001	0.005	<0.001	<0.001	<0.001	<0.001

Note: Different letters (a, b, c, d) represent statistical differences based on Tukey's test (*p* < 0.05, one-way ANOVA).

Abbreviations: ANOVA, analysis of variance; ANPR, apparent net protein retention; FCR, feed conversion ratio; PR, phosphorus retention; PSE, pooled standard error; RAS, recirculating aquaculture system.

^1^ *N* = 3.

^2^One-way ANOVA. Means not sharing any letter are significantly different by the Tukey's HSD-test at the 5% level of significance.

**Table 6:** Whole-body proximate composition of Juvenile shrimp (~0.41 ± 0.01 g) stocked at density of 30 shrimp per tank (35/m^2^) and reared with different levels of protein intake under semi-intensive conditions in tanks of a green water RAS system for 11 weeks.

Protein/ration	Moisture (%)^1^	Protein (%)	Fat (%)	Fiber (%)	Ash (%)	S (%)	P (%)	K (%)	Mg (%)	Ca (%)	Na (%)	Fe (ppm)	Mn (ppm)	Cu (ppm)	Zn (ppm)
D40/100	74.72	76.72	9.33	6.15	10.50	0.75	1.19	1.15	0.26	2.38	0.67	28.60	4.80	157.75	69.67
D40/87.5	74.62	76.87	9.41	5.81	10.76	0.76	1.24	1.21	0.27	2.45	0.69	58.60	5.50	162.00	70.60
D35/100	74.19	75.95	9.56	5.89	10.93	0.75	1.16	1.20	0.26	2.34	0.68	29.65	4.10	151.25	69.08
D35/114.3	74.33	76.20	9.48	5.74	11.20	0.74	1.18	1.17	0.26	2.48	0.69	31.75	4.25	155.75	69.53
D30/100	74.36	76.88	9.11	5.96	10.26	0.72	1.25	1.21	0.27	2.35	0.70	34.55	4.30	166.00	68.58
D35/133.3	73.89	76.55	9.70	6.14	11.22	0.73	1.18	1.16	0.25	2.42	0.71	21.25	3.92	155.50	69.15
D25/100	75.21	78.38	6.96	5.72	11.20	0.71	1.23	1.23	0.26	2.37	0.68	25.65	4.72	159.25	67.62
D25/160	74.16	76.75	8.12	6.04	10.57	0.71	1.14	1.17	0.25	2.33	0.69	26.32	3.88	156.75	68.20
PSE	0.9349	0.7957	1.004	0.4941	0.3549	0.0159	0.0483	0.0382	0.0104	0.2227	0.0285	16.20	0.5634	13.40	1.913
*p*-value^2^	0.670	0.070	0.014	0.862	0.047	0.086	0.166	0.386	0.752	0.977	0.901	0.199	0.141	0.922	0.667

*Note:* Presented on a dry matter basis as means for each treatment.

Abbreviations: ANOVA, analysis of variance; PSE, pooled standard error; RAS, recirculating aquaculture system.

^1^Reported on as is basis.

^2^One-way ANOVA, means not sharing any letter are significantly different by the Tukey's HSD-test at the 5% level of significance.

## Data Availability

The data that support the findings of this study are available on request from the corresponding author.
